# Exploring essential oil-based bio-composites: molecular docking and *in vitro* analysis for oral bacterial biofilm inhibition

**DOI:** 10.3389/fchem.2024.1383620

**Published:** 2024-07-17

**Authors:** Niamat Ullah, Syed Zia Ul Hasnain, Rabia Baloch, Adnan Amin, Aygun Nasibova, Dragica Selakovic, Gvozden Luka Rosic, Sokhib Islamov, Nasibakhon Naraliyeva, Nidal Jaradat, Afat O Mammadova

**Affiliations:** ^1^ Natural Products Research Lab, Gomal Centre of Pharmaceutical Sciences, Faculty of Pharmacy, Gomal University, Dera Ismail Khan, Pakistan; ^2^ Department of Pharmacognosy, Faculty of Pharmacy, Bahauddin Zakariya University, Multan, Pakistan; ^3^ Allama Iqbal Teaching Hospital, Dera Ghazi Khan, Pakistan; ^4^ Department of Biophysics and Biochemistry, Baku State University, Baku, Azerbaijan; ^5^ Institute of Radiation Problems, Ministry of Science and Education Republic of Azerbaijan, Baku, Azerbaijan; ^6^ Department of Physiology, Faculty of Medical Sciences, University of Kragujevac, Kragujevac, Serbia; ^7^ Department of Technology of Storage and Processing of Agricultural Products, Tashkent State Agrarian University, Tashkent, Uzbekistan; ^8^ Department of Botany, Andijan State University, Andijan, Uzbekistan; ^9^ Department of Pharmacy, Faculty of Medicine and Health Sciences, An-Najah National University, Nablus, Palestine; ^10^ Baku State University, Baku, Azerbaijan

**Keywords:** biofilm, bio-composite, molecular docking, essential oil, computational investigations

## Abstract

Oral bacterial biofilms are the main reason for the progression of resistance to antimicrobial agents that may lead to severe conditions, including periodontitis and gingivitis. Essential oil-based nanocomposites can be a promising treatment option. We investigated cardamom, cinnamon, and clove essential oils for their potential in the treatment of oral bacterial infections using *in vitro* and computational tools. A detailed analysis of the drug-likeness and physicochemical properties of all constituents was performed. Molecular docking studies revealed that the binding free energy of a Carbopol 940 and eugenol complex was −2.0 kcal/mol, of a Carbopol 940-anisaldehyde complex was −1.9 kcal/mol, and a Carbapol 940-eugenol-anisaldehyde complex was −3.4 kcal/mol. Molecular docking was performed against transcriptional regulator genes 2XCT, 1JIJ, 2Q0P, 4M81, and 3QPI. Eugenol cinnamaldehyde and cineol presented strong interaction with targets. The essential oils were analyzed against *Staphylococcus aureus* and *Staphylococcus epidermidis* isolated from the oral cavity of diabetic patients. The cinnamon and clove essential oil combination presented significant minimum inhibitory concentrations (MICs) (0.0625/0.0312 mg/mL) against *S. epidermidis* and *S. aureus* (0.0156/0.0078 mg/mL). In the anti-quorum sensing activity, the cinnamon and clove oil combination presented moderate inhibition (8 mm) against *Chromobacterium voilaceum* with substantial violacein inhibition (58% ± 1.2%). Likewise, a significant biofilm inhibition was recorded in the case of *S. aureus* (82.1% ± 0.21%) and *S. epidermidis* (84.2% ± 1.3%) in combination. It was concluded that a clove and cinnamon essential oil-based formulation could be employed to prepare a stable nanocomposite, and Carbapol 940 could be used as a compatible biopolymer.

## 1 Introduction

Oral bacterial infections in diabetic patients are fairly common (Shillitoe et al., 2012). Poor glycemic control facilitates increased and diversified microbial growth in the oral cavity (Xiao et al., 2020), which results in an imbalance in oral microbiota (Al-Janabi, 2023). Gram-positive bacteria, including *Staphylococcus aureus* and *Staphylococcus epidermidis*, are the most common bacteria in the Staphylococci genus responsible for infections in clinical settings (Balachander and Alexander, 2021). Although most *Staphylococci* species are thought to be part of the natural flora, under specific conditions, they can become opportunistic pathogens that can generate a variety of virulence factors (Tong et al., 2015). Various investigations have confirmed the occurrence of *Staphylococci* oral flora; however, they are considered transient members of oral microbiota (McCormack et al., 2015). *S. aureus* and *S. epidermidis* are mainly reported in older people (Murdoch et al., 2004), denture wearers (Tawara et al., 1996), or patients with periodontitis (Loberto et al., 2004). Both strains possess several virulent genes that may be present as distractive loci or as genetic elements (Cheung et al., 2021), including arginine catabolic mobile elements, which give the bacteria the ability to resist some heavy metals, particularly copper ions, and also make it easier for the bacteria to colonize the skin and mucous membranes (Al-Jabri et al., 2021). *S. aureus* and *S. epidermidis* create an extracellular polymeric substance (EPS) that allows the bacteria to settle at the infection site and form a biofilm. EPS acts as a physical barrier to external stress and promotes the growth and maturation of microorganisms (Nguyen et al., 2020). Detachment, the last phase, releases single cells to encourage the spread of biofilm clusters to distant areas (Bertoglio et al., 2018). During the development of biofilm, different soluble factors are produced, including proteins, eDNA, exopolysaccharide, polysaccharide intercellular adhesion (PIA), carbohydrates, teichoic acids, and surfactants (Le et al., 2018). Cell-to-cell signaling by quorum sensing systems also plays an important role in virulent pathogens associated with biofilm formation (Kaur et al., 2021). The main challenge in the oral cavity is the development of bacterial biofilms that limit the permeability of drug moieties to the target site, thus leading to the development of antimicrobial resistance and treatment failure (Dagli and Dagli, 2014; Prestinaci et al., 2015). Thus, there is a great need to investigate new drug moieties to solve this major health concern.

Essential oils (EOs) are gaining the attention of researchers in medical science due to their significant biological activities, high penetration power, and less toxic effects (Chouhan et al., 2017). Essential oils are hydrophobic and evaporated at room temperature (Dhifi et al., 2016). These are mainly comprised of low molecular weight compounds, including monoterpenes and phenolic compounds (Gheorghita et al., 2022). Since ancient times, EOs, including clove oil, have been used in oral hygiene as anti-inflammatory and antimicrobial agents (Saleem et al., 2023). The significant antimicrobial activities of EOs are mainly due to their interference with bacterial membranes due to their hydrophobic nature, which affects cellular structures, and their efflux pump, enzymatic inhibition (β-lactamase), and strong antioxidant properties (Devi et al., 2010; Oliveira et al., 2022). Improved essential oil delivery at the target site can be efficiently achieved through advanced drug delivery systems, including nanocomposites (Guidotti-Takeuchi et al., 2022). These nanocomposites invade the EO molecules and not only protect them from light but also limit their evaporation and offer an efficient delivery at the target site (Joye et al., 2016). The bio-composite materials, including Carbapol 940, are advantageous in achieving efficient drug delivery goals and a high safety profile (Varaprasad et al., 2014). However, weak binding forces between the EOs and bio-composite materials may interfere with the overall formulation.

Modern technology has proposed several tools for new drug discovery and development. The use of computation methods can be a valuable tool for predicting several features of drug likeness, absorption, distribution, metabolism, excretion (ADME) properties, bioavailability, and safety profiling. During drug designing, scientists can make structural changes in molecules with the aim of modifying ADMET (ADME and toxicity) and bioactivity features (van de Waterbeemd and Gifford, 2003). The *in silico* methods offer rapid, easy, and reliable predictions regarding drug–target interaction. Molecular docking is the most common computational structure-based drug design (SBDD) method and has been widely used since the early 1980s (Stanzione et al., 2021). It is the tool of choice when the three-dimensional (3D) structure of the protein target is available, and it mainly helps to understand and predict molecular recognition, both structurally (i.e., finding possible binding modes) and energetically (i.e., predicting binding affinity) (Stanzione et al., 2021). Molecular docking was originally designed to be performed between a small molecule (ligand) and a target macromolecule (protein) (Pawar and Rohane, 2021). However, in the last decade, there has been a growing interest in protein–protein docking, nucleic acid (DNA and RNA)–ligand docking, and nucleic acid–protein–ligand docking. (Rahman et al., 2022).

In formulation design, component interactions with polymers are considered very important because they can affect the permeability and bioavailability of drug molecules. Therefore, we aimed to investigate EO-based nanocomposite components analysis by using molecular dynamics, docking, and *in vitro* analysis to determine the efficacy of the formulation.

## 2 Materials and methods

### 2.1 Chemicals and strains

Essential oils (cardamom, cinnamon, and clove) were extracted in the Natural Products Research Laboratory at Gomal University D.I. Khan. The bacterial growth media used included Tryptic Soy Broth (Hi Media, Mumbai, India), nutrient agar (Hi Media, Mumbai, India), and Luria-Bertani Broth (LB) (Oxoid, Hampshire, United Kingdom). The standard compounds, including eugenol (Fluka, Riedstr, Germany), cinnamaldehyde (Sigma Aldrich, St. Louis, MO, United States), and resazurin (Sigma Aldrich, St. Louis, MO, United States), were purchased commercially.

### 2.2 Physicochemical *in silico* analysis

The drug likeness and ADMET analyses of the essential oils were determined by using different online tools like pkCSM ADMET and SWISS ADME (Douglas et al., 2015; Daina et al., 2017; Amin et al., 2020). The simplified molecular input line entry system (SMILES) was used to load the tested compounds on the input path of the above-mentioned online computational tools, and data were generated. All results were recorded, and interpretations were framed accordingly.

### 2.3 Polymer docking

The structures of Carbapol 940, eugenol, and anisaldehyde were downloaded from PubChem. Energy minimization of all generated structures was carried out using YASARA Structure software (Karieger and Vriend, 2014). The structures of nanocomposites, including Carbapol 940, eugenol, and anisaldehyde, were considered as alternative receptors (host) and ligands (guest) to obtain the stable emulsion complex. AutoDock Vina v 4.2.6 was used for molecular docking calculations in PyRx, in which the grid box was set to cover the entire component to ensure that all possible interactions with the system were searched (Dallakyan and Olsan, 2015). Discovery Studio Visualizer was used for the visualization and graphical representations of all complexes (Trott and Olson, 2010).

### 2.4 Ligand docking (MD studies)

AutoDock Vina v 4.2.6 was used for molecular docking. The Protein Data Bank (PDB) was used to obtain the X-ray crystallographic structure of the transcriptional regulators 2Q0J, 3QP1, 1JIJ, 2XCT, and 4M8I in PDB format. These target genes were further aligned for molecular docking in Discovery Studio 2.0 for the removal of water and H atoms and the addition of charges. The ligand 3D structures were collected from the PubChem database. The active pocket determination was performed by using the CASTp 3.0 tool. Molecular docking was performed by AutoDock v 4.2.6 using the Lamarckian genetic algorithm. Both the ligand and target were further processed for torsion, Kollman charges, and other required alignments. Finally, a command prompt was used for molecular docking. The best-docked molecules with the highest free binding energy [ΔG] were examined using Ligplot + Accelrys DS Visualizer 2.0 and PyMOL. The generated poses were classified according to their root mean square deviation (RMSD) values (Qaiserani et al., 2021).

### 2.5 Biological evaluation

#### 2.5.1 Minimum inhibitory concentration (MIC)

The minimum inhibitory concentration of the essential oil was determined by the broth dilution method with slight modification (Ullah et al., 2019). A 50-µL aliquot of nutrient broth was added to each well in a 96-well microplate, and 50 µL of the test sample was added to this with serial dilution. Finally, 50 µL of the test strain was added to each well, and the 96-well microplate was incubated for 24 h at 37°C. Afterward, an aliquot of 40 μL of resazurin solution (0.015%) was added to each well and incubated for 1 h. The color changes in the 96-well microplates were recorded.

#### 2.5.2 Anti-biofilm assay

The antibiofilm activity of the test sample was determined by using a modified method (Rafey et al., 2021). In brief, 24 h-old bacterial cultures (200 μL, adjusted with 0.5 McFarland) and 50 μL of the test sample were added to each well in a 12-well plate and incubated for 24 h at 37°C. Bacterial growth was measured using a spectrophotometer (λ 592 nm). For quantification, crystal violet staining of the biofilm was adopted (in the 12-well plates), followed by the addition of 95% ethanol to the stained cells. Finally, the absorbance at 592 nm was measured and determined using the following equation:

$$\text{Inhibition}\left(\%\right)=\left[1\;‐\;\left(\text{Absorbance}\;\text{of}\;\text{sample}\;/\text{Absorbance}\;\text{of}\;\text{control}\right)\right]\times 100.$$

#### 2.5.3 Anti-quorum sensing

The anti-quorum sensing activity of the test sample was determined by using biomarker strain *C. violaceum* (Amin et al., 2020). A 24 h-old strain of the *Chromobacterium violaceum* (1/100 ratio) was steaked onto the LB agar, and sterilized 6 mm filter paper discs were placed in the center of the Petri dishes. A 15 μL aliquot of the test sample was loaded on filter paper discs and allowed to dry for 30 min. The plates were placed in the incubator at 30°C for 24 h. After 24 h, the zone of inhibition was measured, and the results were recorded.

#### 2.5.4 Violacein quantification assay

The violacein quantification was performed by a standard procedure (Amin et al., 2020). Briefly, a 200 μL aliquot of *C violaceum* (OD = 0.4 OD at 600 nm) along with 25 µL of the test sample was added in a 96-well microplate. The 96-well microplate was then incubated at 30°C for 24 h. Then, the decrease in the violacein pigment synthesis was detected by determining the absorbance at 585 nm. The following formula was used to determine the % violacein inhibition:

$$\text{Violacein inhibition }\left(\%\right)=\left[1\;‐\;\left(\text{Absorbance of sample}/\text{Absorbance of control}\right)\right]\times 100.$$

## 3 Results

### 3.1 Polymer docking

**FIGURE 1:**
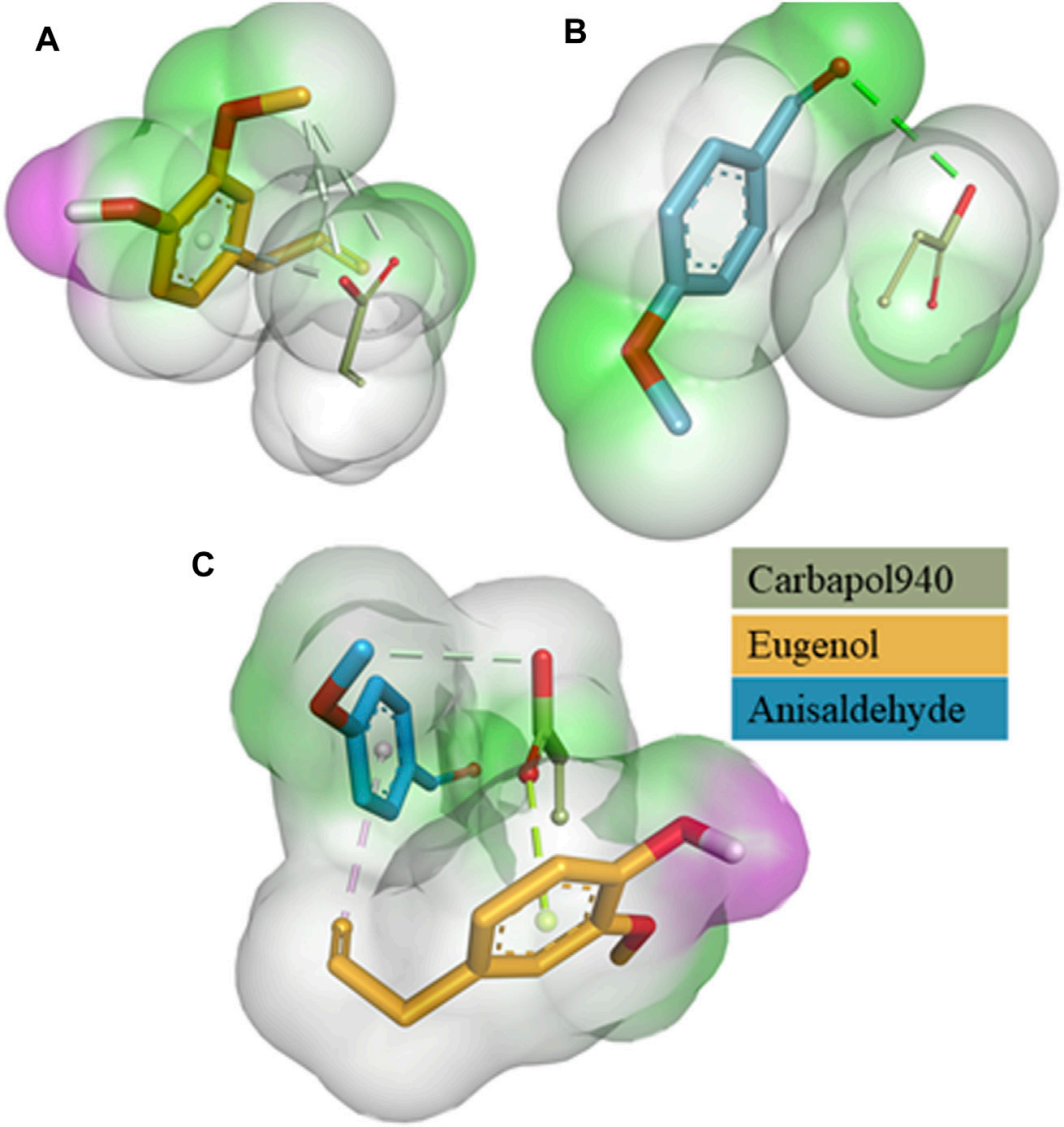
3D surface and binding interaction representation of Carbapol 940-Eugenol Complex **(A)**. 3D surface and binding interaction representation of Carbapol 940-Anisaldehyde complex **(B)** and 3D surface and binding interaction representation of Carbapol 940-Eugenol-Anisaldehyde complex **(C)**.

The relative binding free energies between the clove oil (eugenol), cinnamon oil (cinnamaldehyde), and the gelling agent (Carbopol 940) molecules were determined using AutoDock Vina, as indicated in (Table 1). Binding interactions are depicted in Figure 1. The molecular docking investigations revealed that the proposed formulations might be stable.

**TABLE 1:** Interaction analysis of diverse formulation systems.

S. No	Composite ingredient	(kcal/mol)^a^
1	Carbapol 940-eugenol	−2.0
2	Carbapol 940-anisaldehyde	−1.9
3	Carbapol 940-eugenol-anisaldehyde	−3.4

^a^ Binding energies.

### 3.2 Ligand docking

**FIGURE 2:**
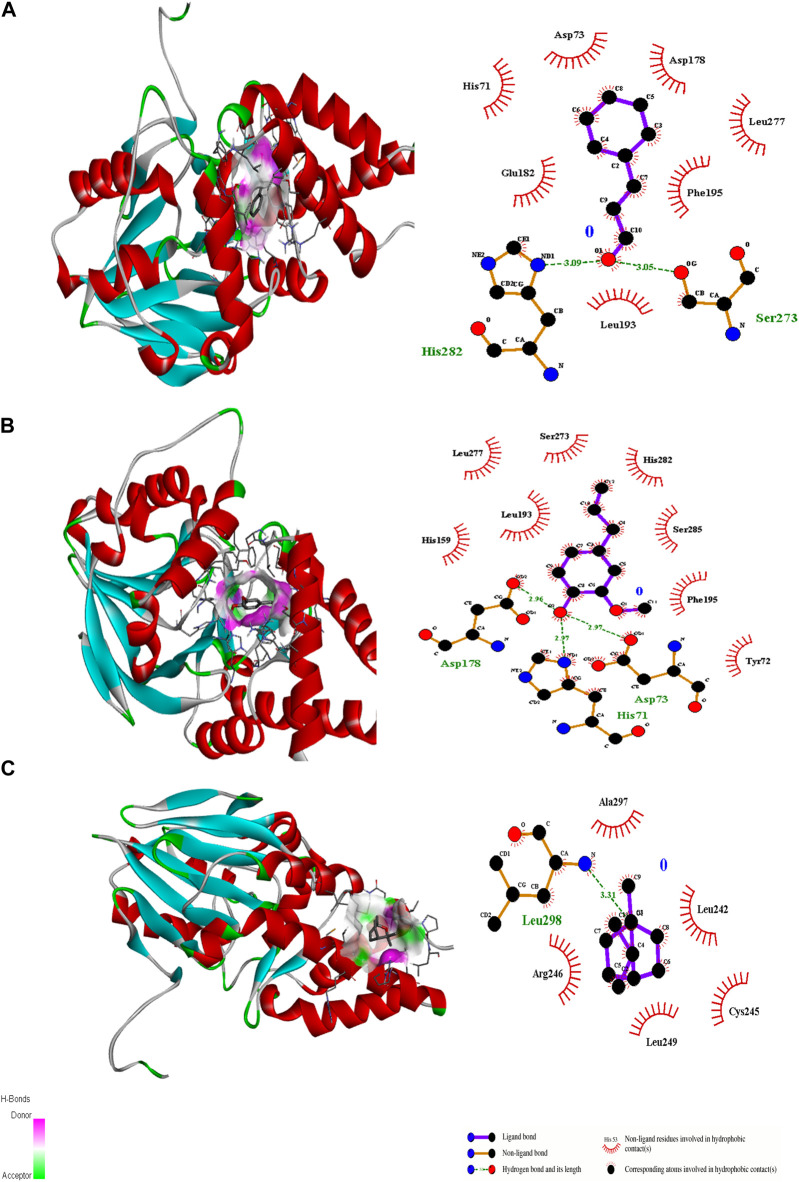
3D interactions of Cinnamaldehyde **(A)** (Pose 1), eugenol **(B)** (Pose 2) and cineol **(C)** (Pose 1) with 2Q0J binding site.

**FIGURE 3:**
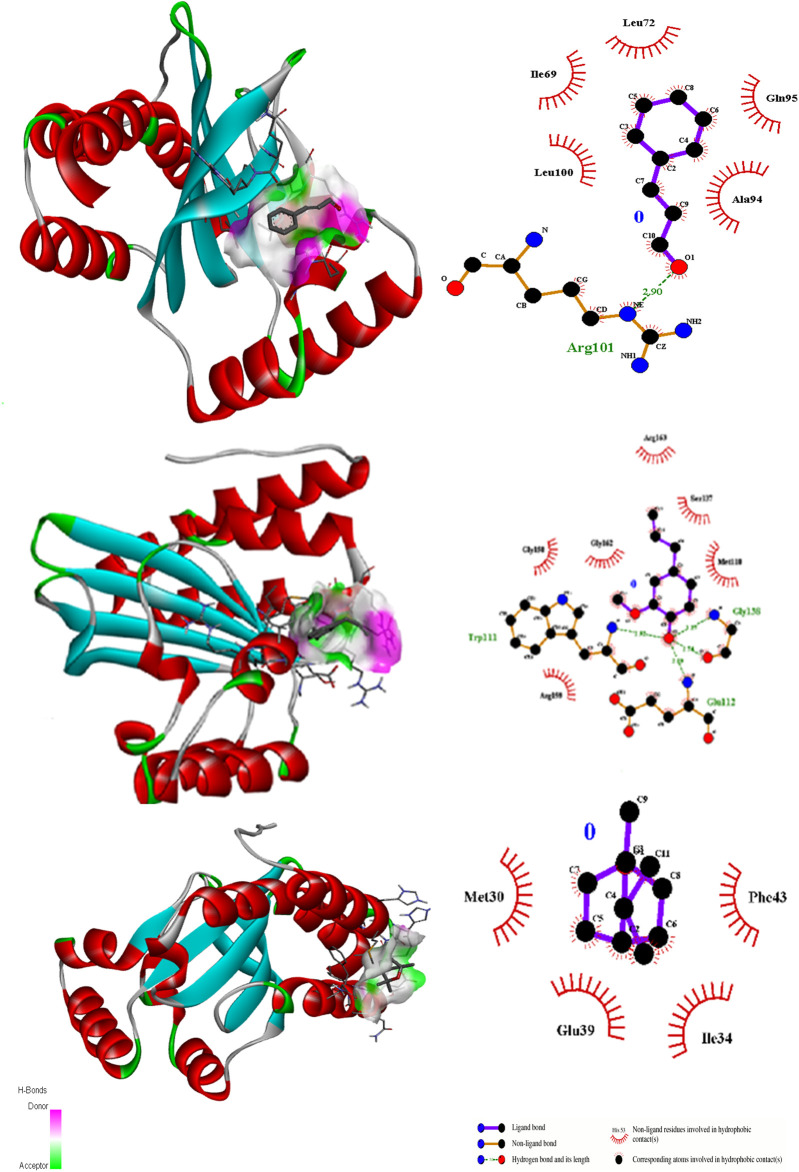
3D interactions of Cinnamaldehyde **(A)** (Pose 1), eugenol **(B)** (pose 2) and cineol **(C)** (Pose 1) with 3QP1 binding site.

**FIGURE 4:**
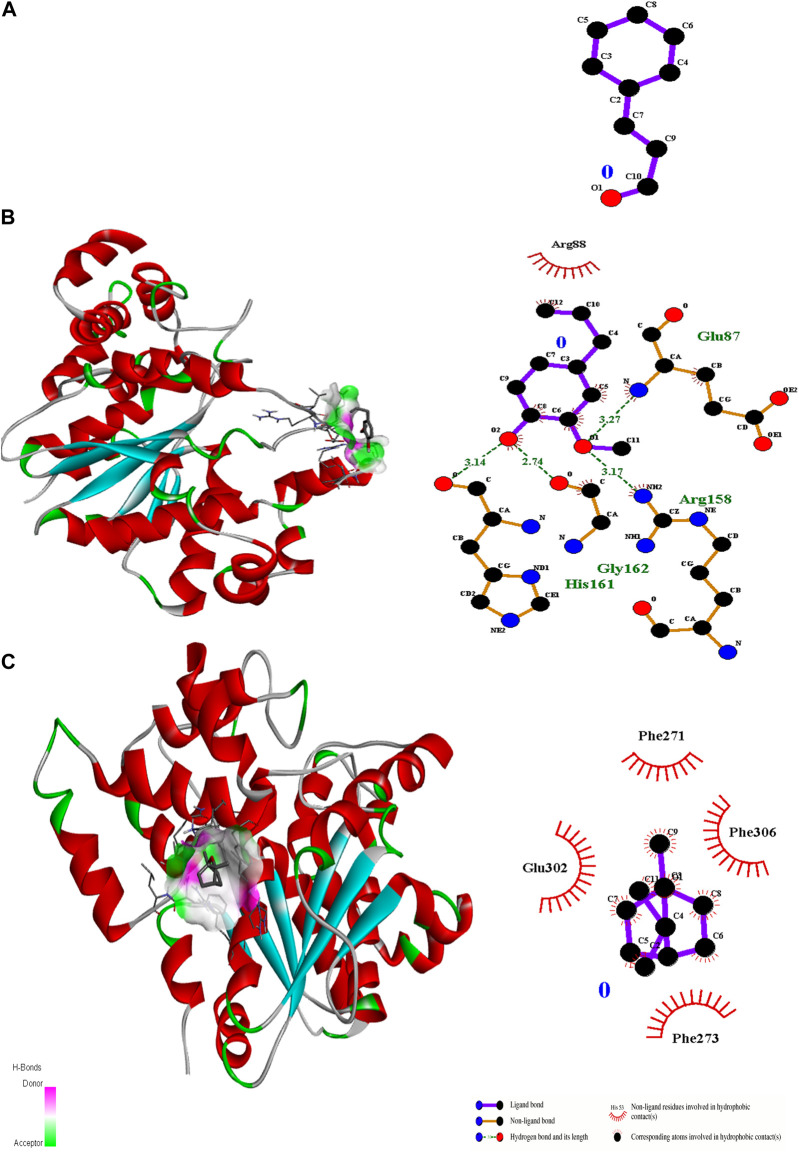
3D interactions of Cinnamaldehyde **(A)** (Pose 1), eugenol **(B)** (pose 1) and cineol **(C)** (Pose 1) with 1JIJ binding site.

**FIGURE 5:**
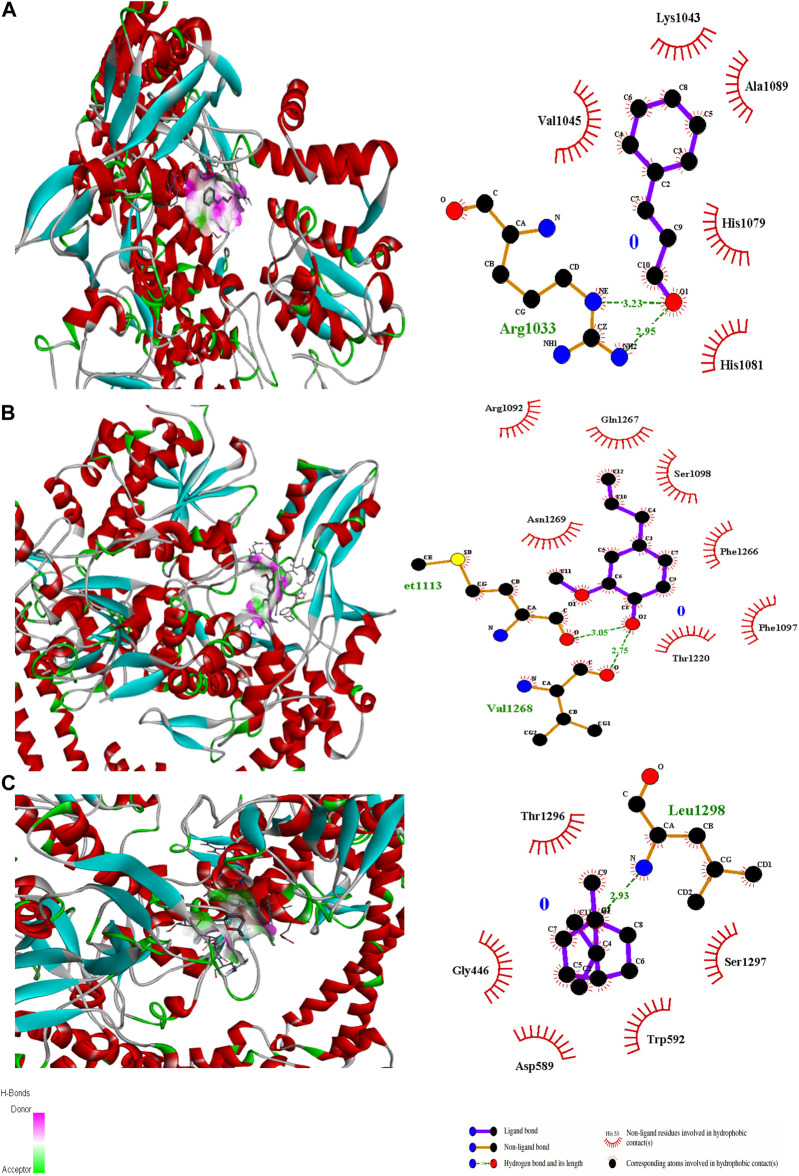
3D interactions of Cinnamaldehyde **(A)** (Pose 8), eugenol **(B)** (pose 1) and cineol **(C)** (Pose 1) with 2XCT binding site.

**FIGURE 6:**
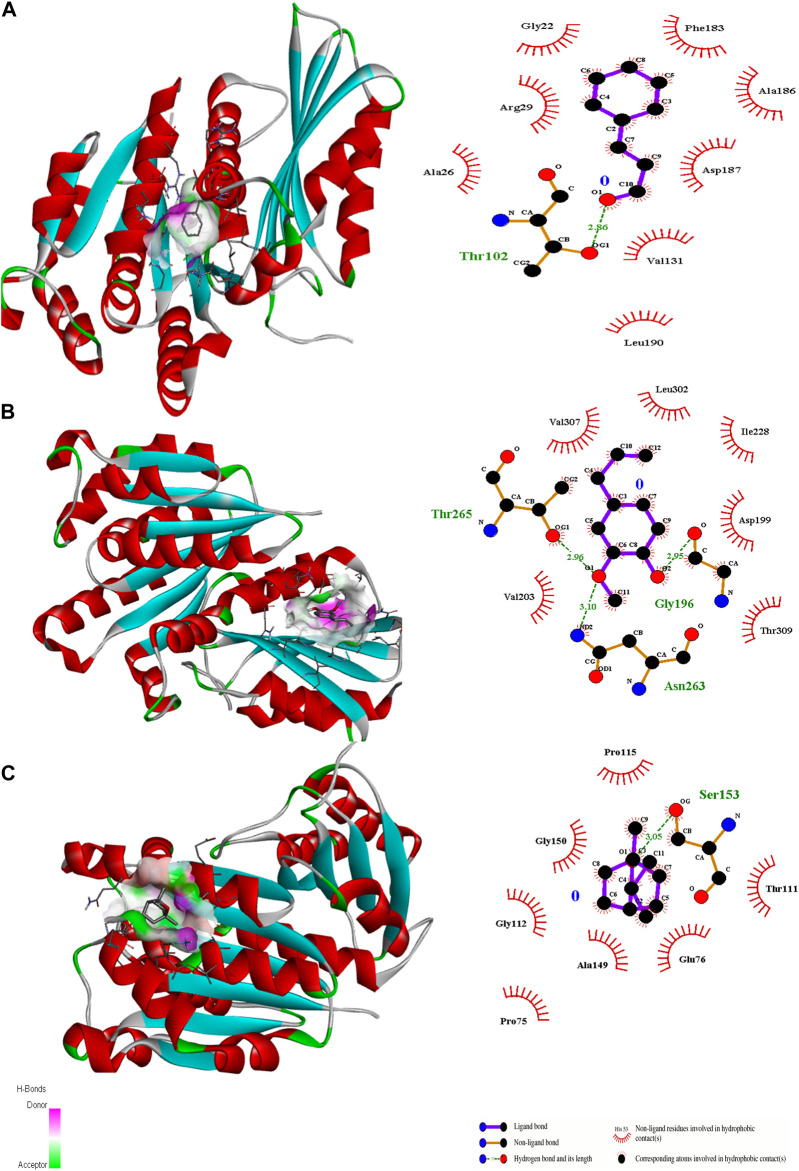
3D interactions of Cinnamaldehyde **(A)** (Pose 1), eugenol **(B)** (pose 1) and cineol **(C)** (Pose 6) with 2Q0J binding site.

Ligand docking (cinnamaldehyde, eugenol, and cineol) was performed with transcriptional regulator 2Q0J (*Pseudomonas* quinolone signal response protein PqsE), anti-quorum sensing regulator gene 3QP1, 1JIJ (*S. aureus* tyrosyl-tRNA synthetase), 2XCT (*S.aureus* topoisomerase-II DNA gyrase), and 4M8I (the bacterial cytoskeletal division protein filamentous temperature-sensitive mutant Z (FtsZ)). Results of molecular docking revealed strong H-bonding interactions within the active pocket of all transcription regulator genes with eugenol and cinnamaldehyde (Table 2) with significant free binding energies (2 ΔG (kJ mol‒1)). The molecular docking interaction analysis results with amino acid residues are presented in Figures 2–6.

**TABLE 2:** Docking analysis of major essential oil components.

Sample	ΔG (kJ mol^‒1^)	Pose No.	H bonds	Amino acids interacting through an H bond	Amino acids with other bonds
2Q0J					
Cinnamaldehyde	−6.0	2	2	Ser273 and His282	Leu193, Glu182, His71, Asp73, Asp73, Asp178, Leu277, and Phe195
Eugenol	−6.2	2	3	Asp73, His71, and Asp178	Tyr72, His159, Leu193, Leu277 Ser273, His282, Ser285, and Phe195
Cineol	−4.6	1	1	Leu298	Leu249, Arg246, Ala297, Leu 242, and Cys245
3QP1					
Cinnamaldehyde	−4.4	1	1	Arg101	Ala94, Gln95, Leu72, Ile69, and Leu100
Eugenol	−5.0	6	3	Trp111, Gly128, and Gla112	Arg159, Gly158, Gy162, Arg163, Ser137, and Met110
Cineol	−4.4	1	0	0	Phe43, Ile34, Glu39, and Met30
1JIJ					
Cinnamaldehyde	NIL				
Eugenol	−3.1	1	3	Arg158, Gly162, and His161	Arg88
Cineol	−5.4	1	0	0	Phe273, Glu302, Phe271, and Phe306
2XCT					
Cinnamaldehyde	−4.8	8	1	Arg1033	His1081, His1079, Ala1089, Lys1043, and Val1045
Eugenol	−5.9	1	2	Val1268 and Met1113	Asn1269, Arg1092, Gln1267, Ser1098, Phe1266, Phe1097, and Thr1220
Cineol	−5.4	1	1	Leu1298	Asp589. Gly446, Thr1296, Ser1297, and Trp592
4M8I					
Cinnamaldehyde	−5.9	1	1	Thr102	Leu190, Val131, Asp187, Ala186, Phe183, Gly22, Arg29, and Ala26
Eugenol	−5.7	1	3	Asn263, Gly196, and Thr265	Val203, Val307, Leu302, Ile228, Asp199, and Thr309
Cineol	−4.9	6	1	Ser153	Ala149, Pro75, Gly112, Gly150, Pro115, Thr111, and Glu76

### 3.3 Drug likeness

The drug-likeness studies were performed using the Molinspiration tool following Lipinski’s rule of five. The major constituents of essential oils identified by gas chromatography-mass spectroscopy (GCMS) data (Rafey et al., 2021) were screened for drug likeness. It was evident from the computational data that all tested compounds showed good compliance with drug likeness according to Lipinski’s rule (Table 3). Thus, all tested compounds could be drug candidates.

**TABLE 3:** Lipinski’s rule of five application data.

Sample	MW	Relative amount (%)	Log p	H donor	H acceptor	Violation
Cinnamaldehyde	132.16	62	2.48	0	1	0
Eugenol	164.20	85	2.10	1	2	0
Linalool	154.25	25	3.21	1	1	0
Cineol	154.24	40	2.72	0	1	0

MW (molecular weight<500 kDa); Log p(<5); H donor(H bond donor <5); H acceptor(H-bond acceptor(<10); Violations (No. of violations).

### 3.4 Drug-likeness score

**FIGURE 7:**
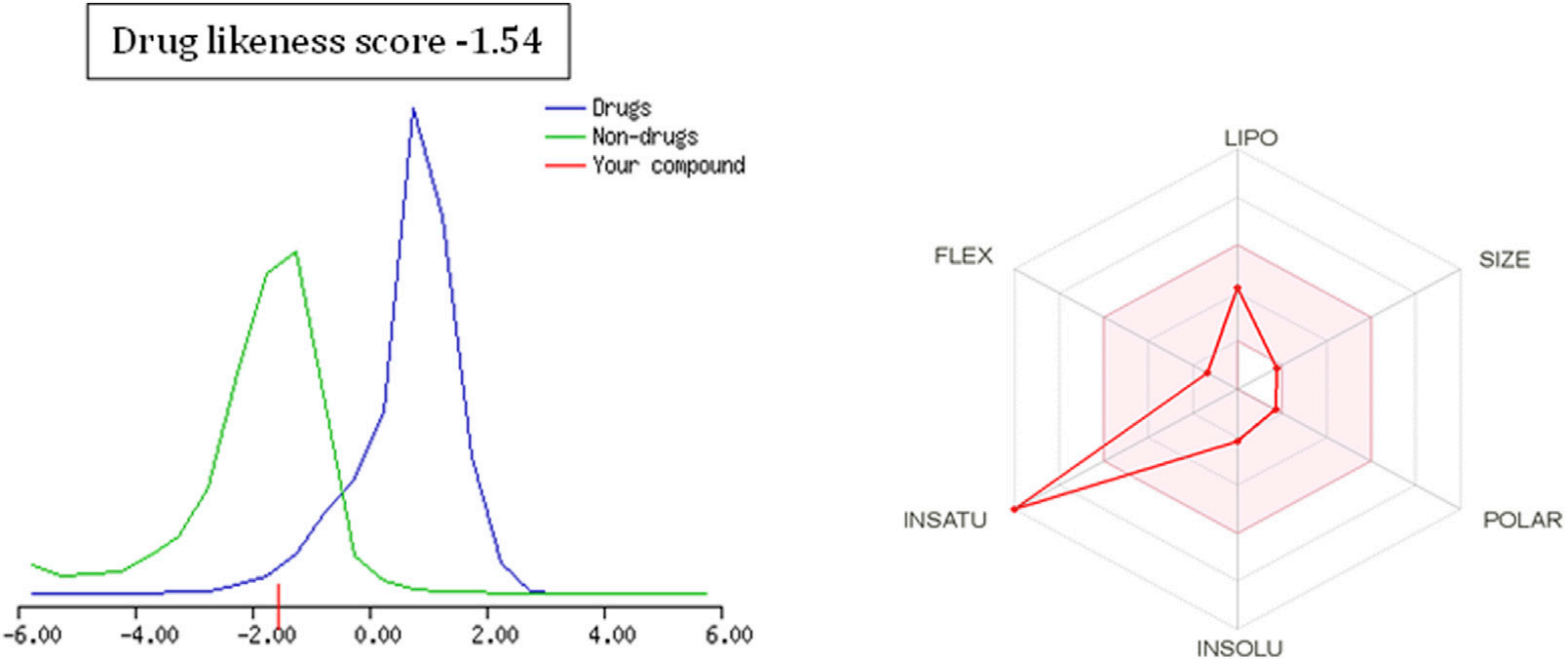
Drug-likeness score and bioavailability radar prediction for cinnamaldehyde.

**FIGURE 8:**
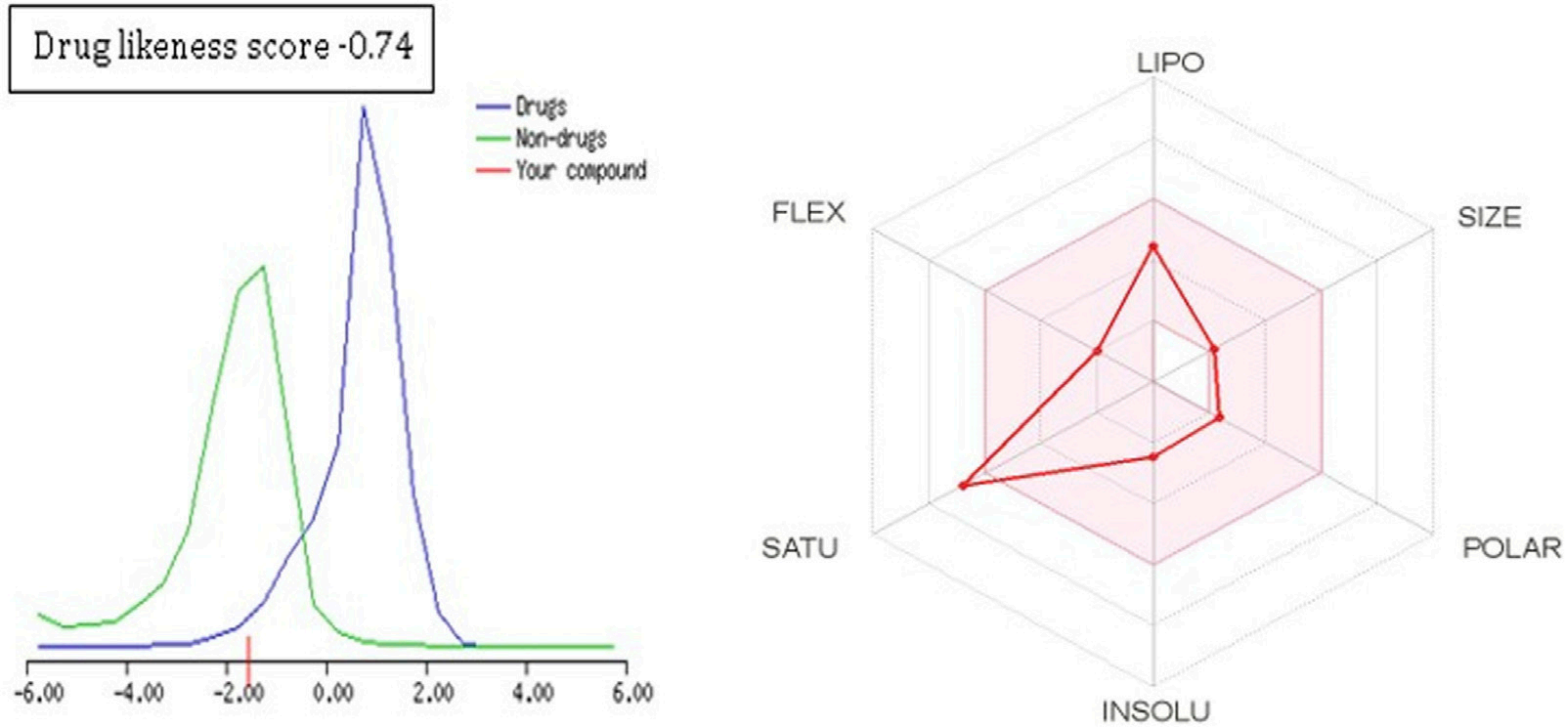
Drug-likeness score and bioavailability radar prediction for eugenol.

**FIGURE 9:**
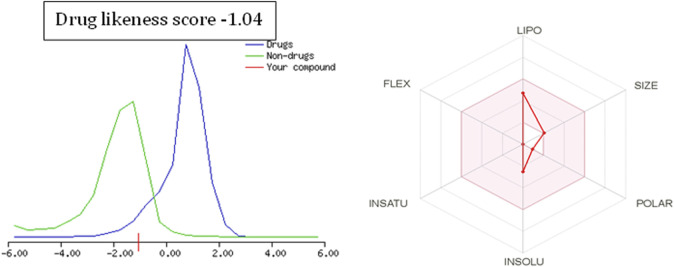
Drug-likeness score and bioavailability radar prediction for cineol.

**FIGURE 10:**
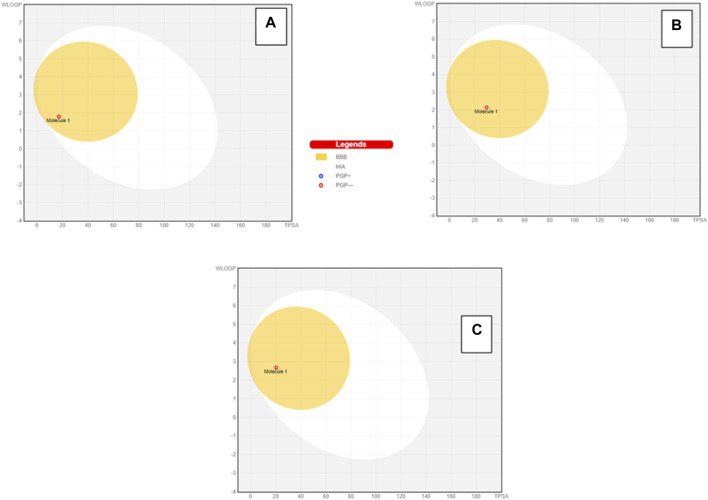
BOILED-Egg model prediction of GIT absorption and brain bioavailability of cinnamaldehyde **(A)**, eugenol **(B),** and cineol **(C)**.

To further extend the scope of the investigation, the drug-likeness score was determined using Molsoft’s chemical fingerprints model (Kamoutsis et al., 2021). The bioavailability was predicted using Bioavailability Radar (SWISS ADME) and the Brain Or IntestinaL EstimateD permeation (BOILED-Egg) model (SWISS ADME) (Kamoutsis et al., 2021). The cinnamaldehyde presented a slight deviation from the standard value of the drug-likeness score (less than one), whereas all other molecules were within the permissible range (Figures 7–9). Likewise, all molecules were within permissible limits of bioavailability (Figures 7–9) and could cross the blood–brain barrier (Figure 10).

### 3.5 ADMET analysis

The ADMET analysis was performed by using the Molinspiration tool to determine the ADMET attributes of all tested compounds (Table 4). The results of the analysis revealed that all tested molecules are in agreement with the set parameters and could be utilized for oral formulations (Table 4).

**TABLE 4:** ADMET properties of compounds.

Properties	Compounds		
	Cinnamaldehyde	Eugenol	Cineol
TPSA [A°]	17.07	29.46	20.23
Consensus log P_o/w_	1.95	2.25	2.66
Water solubility [log mol/L]	−2.175	−1.504	−2.63
CaCo_2_:permeability [log Papp in 10^−6^ cm/s]	1.634	1.558	1.485
Intestinal absorption [human] [% absorbed]	95.015	93.375	96.505
Skin permeability [log Kp]	−2.355	−1.822 No	−2.437
P-Glycoprotein substrate	No	No	Yes
P-Glycoprotein I inhibitor	No	No	No
P-Glycoprotein II inhibitor distribution	No	No	No
VDss [human, log L/kg]	0.266	0.217	0.491
Fraction unbound [human][fu]	0.3	0.296	0.553
BBB permeability [logBBI]	0.436	0.185	0.368
CNS permeability [log PS]	−1.582	−2.034	−2.972
**Metabolism**			
CYP2D6 substrate	No	No	No
CYP3A4 substrate	No	No	No
CYP1A2 inhibitor	Yes	Yes	No
CYP2C19 inhibitor	No	No	No
CYP2C9 inhibitor	Yes	No	No
CYP2D6 inhibitor	No	No	No
CYP3A4 inhibitor	No	No	No
**Excretion**			
Total clearance [logml/min/kg]	0.203	0.27	1.009
Renal OCT2 substrate	No	No	No
**Toxicity**			
AMES toxicity	No	Yes	No
hERG I inhibitor	No	No	No
hERG II inhibitor	No	No	No
Hepatotoxicity	No	No	No
Skin sensitization	Yes	Yes	Yes

### 3.6 Antimicrobial assays

#### 3.6.1 Determination of MICs

**FIGURE 11:**
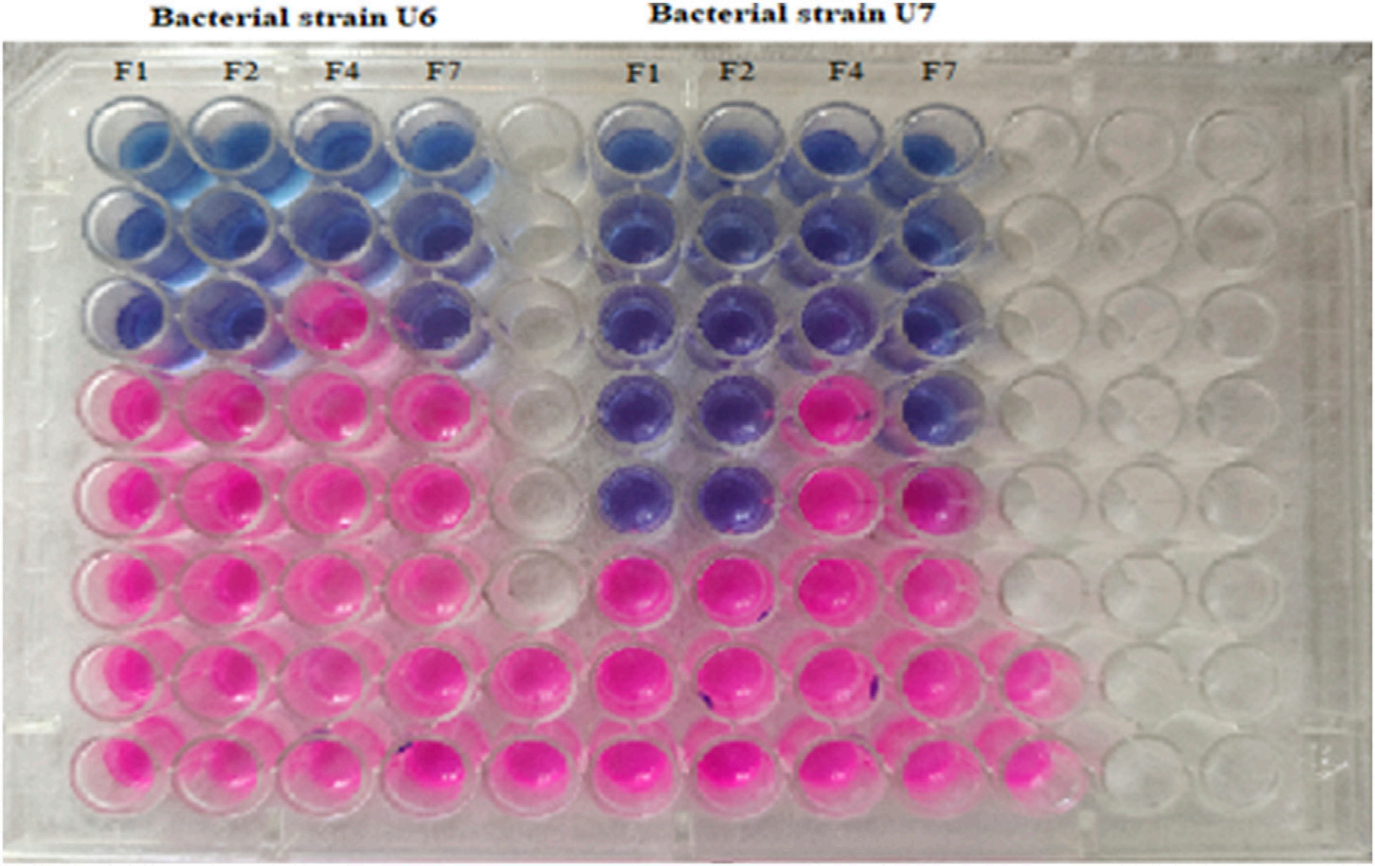
Determination of MIC of diverse combinations of essential oils against tested strains of U6 (*S. epidermidis*) and U7 (*S. aureus*).

The cinnamon, cardamom oil, and clove EOs were tested individually and then mixed in definite ratios and tested for the effects of the different combinations (Table 5). In the individual cases, the clove EO showed the highest MIC (0.024 mg/mL) against *S. epidermidis*, followed by the cinnamon EO (MIC 0.039 mg/mL). In the case of *S. aureus,* the cinnamon EO showed the highest inhibition (0.078 mg/mL), followed by the clove EO (0.097 mg/mL) (Table 6; Figure 11). Based on these findings, it was decided to further process only clove, cinnamon, and cardamom oil to see their combined effect.

**TABLE 5:** Combination of the essential oils.

C. Code	Cinnamon oil (%)	Cardamom oil (%)	Clove oil (%)
F1	1	1	
F2	1		0.5
F3		1	0.5
F4	1	1	0.5

**TABLE 6:** Minimum inhibitory concentration of the individual essential oil.

Essential oil	*S. epidermidis* (MIC mg/mL)	*S. aureus* (MIC mg/mL)
Clove	0.024	0.097
Cinnamon	0.039	0.078
Cardamom	6.25	0.25
Standard^a^	0.24	0.48

^a^ Ciprofloxacin µg/mL.

In the MIC assays of diverse combinations (as explained above), combination F2 comprising cinnamon and clove EOs was chosen for further activity because this mixture showed excellent inhibition of *S. epidermidis* (0.0625/0.0312 mg/mL) and *S. aureus* (0.0156/0.0078 mg/mL) (Table 7). It was concluded that the mixture of clove and cinnamon EOs may have a synergistic effect in the eradication of oral bacteria.

**TABLE 7:** Minimum inhibitory concentration of the different combinations of essential oils.

C. code	Combination^a^	*S. epidermidis* (MIC mg/mL)	*S. aureus* (MIC mg/mL)
F1	Cinnamon oil	0.0625	0.0156
	Cardamom oil	0.0625	0.0156
**F2**	Cinnamon oil	0.0625	0.0156
	Clove oil	0.0312	0.0078
F4	Cardamom oil	0.125	0.0625
	Clove oil	0.0625	0.0312
F7	Cinnamon oil	0.042	0.0208
	Cardamom oil	0.042	0.0208
	Clove oil	0.0208	0.0104
Standard	Ciprofloxacin	0.24	0.48

^a^ Combination ratio 1:1

^b^ µg/mL.

#### 3.6.2 Anti-quorum sensing properties

In this experiment, initially, different concentrations of essential oils were tested for anti-quorum sensing activities, and the N2 formulation presented promising inhibitory zones (8 mm) and significant violacein inhibition (58% ± 1.2%) (Tables 8–9).

**TABLE 8:** Anti-quorum sensing activities of clove and cinnamon essential oils (F1–F7).

C. code	Combination	Concentration (% v/v)	Zone of inhibition (mm)	% Violacein inhibition
F1	Cinnamon	0.5	4	20% ± 1.6%
	Cardamom	0.5		
F2	Cinnamon	0.5	5	24% ± 0.45%
	Clove	0.25		
F4	Cardamom	0.5	0	0
	Clove	0.25		
F7	Cinnamon	0.5	3	12% ± 1.4%
	Cardamom	10.5		
	Clove	0.25		
Standard^a^		20	16	70% ± 0.4%

**TABLE 9:** Anti-quorum sensing activities of clove and cinnamon essential oils (N1–N4)

C. Code	Combination	Conc (% v/v)	Zone of inhibition (mm)	% Violacein inhibition
N1	Cinnamon	1	7 mm	52 ± 1.3%
	Cardamom	1		
N2	Cinnamon	1	8 mm	58 ± 1.2%
	Clove	0.5		
N3	Cardamom	1	1 mm	0%
	Clove	0.5		
N4	Cinnamon	1	6 mm	51 ± 0.08%
	Cardamom	1		
	Clove	0.5		
Standard^a^		20	16	70 ± 1.4%

a Ciprofloxacin (µg/mL).

#### 3.6.3 Antibiofilm properties

Based on the findings of previous experiments, the antibiofilm properties of the essential oil combinations were determined against both tested strains for 72 h. All combinations showed promising antibiofilm activities; however, N2 (84.2% ± 1.3%) and N3 (81.3% ± 0.81%) were effective against *S. epidermidis* for 72 h (Table 10). Likewise, significant inhibition was noticed against *S. aureus*, where promising inhibition was recorded in the case of N2 (82.1% ± 0.21%) and N3 (78.5% ± 0.14%) (Table 11).

**TABLE 10:** Antibiofilm properties of essential oil combinations against *S. epidermidis*.

Time (h)	Antibiofilm activity (%)				
	N1 (%)	N2 (%)	N3 (%)	N4 (%)	Standard*
24	60.3 ± 2.1	62 ± 1.21	66.6 ± 1.3	27.6 ± 1.7	72% ± 1.4%
48	64.1 ± 0.47	69.5 ± 1.5	54.3 ± 0.61	47.4 ± 2.1	72% ± 1.3%
72	79 ± 1.4	84.2 ± 1.3	81.3 ± 0.81	72.7 ± 1.3	86% ± 2.4%

Ciprofloxacin @ 28 μg/mL.

**TABLE 11:** Antibiofilm properties of essential oil combinations against *S. aureus*.

Time (h)	Antibiofilm activity (%)				
	N1 (%)	N2 (%)	N3 (%)	N4 (%)	Standard*
24	64 ± 2.4	70.1 ± 0.21	62.3 ± 0.14	45.1 ± 2.1	70% ± 0.42%
48	62.1 ± 0.74	72.1 ± 1.6	58.2 ± 0.21	54.2 ± 0.41	68% ± 0.62%
72	75 ± 0.12	82.1 ± 0.21	78.5 ± 0.14	59.2 ± 0.63	82% ± 0.81%

Ciprofloxacin @ 28 μg/mL.

## 4 Discussion

Molecular docking is an important modeling approach that gives an idea about the interactions between receptor (host) and ligand (guest). This *in silico* method depicts the ligand binding sites and conformations within a host. Molecular docking simulation gives insight into the orientation of the drug in a binding site (called its “pose”) and also gives an estimation of the binding affinity of the identified pose in the form of a scoring value (Ahmed et al., 2018). The AutoDock Vina algorithm uses a machine learning method that merges the advantages of knowledge-based potentials and empirical scoring functions to calculate the binding energy of a given ligand pose. Ligand docking of cinnamaldehyde, eugenol, and cineol was performed with the transcriptional regulator 2Q0J (*Pseudomonas* quinolone signal response protein PqsE), and interactions were recorded. All ligands showed interaction with the target; however, in the case of eugenol, maximum H-bonding interactions were recorded (3) with pose 2 (−6.2 ΔG (kJ mol^‒1^). The participating amino acids were Asp73, His71, and Asp178, and neighboring amino acids included Tyr72, His159, Leu193, Leu277, Ser273, His282, Ser285, and Phe195 (Table 2; Figure 2). It was concluded that both H-bonding and hydrophobic interactions participate in this case and may contribute towards antibiofilm potential. Another interesting interaction in 2Q0J was recorded in the case of cinnamaldehyde, which showed a better interaction in the case of pose 2 with a binding energy of −6.0 ΔG kJ mol^‒1^. In this case, Ser273 and His282 showed H-bonding interaction whereas hydrophobic interactions were recorded with Leu193, Glu182, His71, Asp73, Asp73, Asp178, Leu277, and Phe195.

In the case of the anti-quorum sensing regulator gene 3QP1, eugenol showed the best fit in the active pocket with pose 3 (−5.0 ΔG (kJ mol^‒1^). The H-bonding was contributed by Trp111, Gly128, and Gla112, and other non-H bonding interactions were contributed by neighboring amino acids, including Arg159, Gly158, Gy162, Arg163, Ser137, and Met110 (Table 2; Figure 3). Most importantly, in the case of cinnamaldehyde, only one H-bonding interaction was recorded with Arg101 amounting to low binding energy (−4.0 ΔG (kJ mol^‒1^), and no interaction was seen in the case of cineol. This predicts that eugenol may have anti-quorum sensing activities.

In the case of transcriptional regulator 1JIJ (*S. aureus* tyrosyl-tRNA synthetase), no interaction was recorded with cinnamaldehyde or cineol, which shows no or less inhibition potential of these compounds. In contrast, the eugenol showed best fit in the active pocket of the target site (1JIJ). The best fitting occurred with pose 1 and had low binding energy (−3.1 ΔG (kJ mol^‒1^), three H-bonding interactions, comprising Arg158, Gly162, and His161, and the neighboring amino acid was Arg88, involved in hydrophobic interactions (Table 1; Figure 4). Docking of compounds on the active pockets of transcriptional regulator 2XCT (*S.aureus* topoisomerase-II DNA gyrase) revealed that all tested compounds had low levels of interaction comprising one H-bond interaction (Table 1; Figure 5). However, the binding energy of eugenol was significant (−5.9 ΔG (kJ mol^‒1^) with pose 1 and showed interactions with Val1268 and Met1113. In this case, the hydrophobic interactions were seen with Asn1269, Arg1092, Gln1267, Ser1098, Phe1266, Phe1097, and Thr1220. In the case of transcriptional regulator 4M8I (the bacterial cytoskeletal division protein filamentous temperature-sensitive mutant Z (FtsZ)) in eugenol, the best fitting was noticed in pose 1 (−5.7 ΔG (kJ mol^‒1^), where three H-bond interactions included Asn263, Gly196, and Thr265 (Table 2; Figure 6). The hydrophobic interactions were contributed by amino acids, including Val203, Val307, Leu302, Ile228, Asp199, and Thr309. In the case of cinnamaldehyde, the best fitting was seen with pose 1 (−5.1 ΔG (kJ mol^‒1^), and only one amino acid, Thr102, contributed to H-bond formation, which shows a lesser participation of this compound in inhibition.

Molecular docking investigations were performed to investigate the stability of the major components of the formulation and active essential oils. The binding free energies between Carbapol 940 (host) and eugenol and anisaldehyde molecule (guests) estimated the strength of the interactions between them. Tighter interactions between the drug molecules and gelling agent might lead to a stable emulsion and may result in a more sustained drug release profile than looser interaction/binding (Hameed et al., 2020; Khan et al., 2021). It was also apparent from the binding free energies table that the emulgel form has a lower binding affinity than the co-ligand form. For example, mono-ligand complexes, including Carbapol 940-eugenol and Carbapol 940-anisaldehyde (−2.4 kcal/mol) complexes, were found to have less binding affinity than Carbapol 940-eugenol-anisaldehyde (−3.4 kcal/mol). It was evident that bio-composite Carbapol 940 and EO components were compatible with each other and could offer a stable nanocomposite, as reported earlier (Lu et al., 2021). Furthermore, in the case of EOs, nanocomposite-based formulations are advantageous compared to conventional dosage forms because they limit the EO evaporation and allow enhanced drug delivery (Varaprasad et al., 2014).

The empirical range of drug-likeness scores is −1 to +1. In the case of cinnamaldehyde, the drug-likeness score is −1.54, which is out of range, whereas eugenol and cineol have −0.74 and −1.04, are within range and thus fulfill the criteria of drug-likeness (Figure 6). Bioavailability Radar is another helpful tool for quickly seeing the drug likeness of a molecule. The pink region reflects the best range for oral bioavailability for each particular property. [Lipophilicity (LIPO): XLOGP3 ranges from 0.7 to +5.0; Polarity (POLAR): A topological polar surface area (TPSA) between 20 and 130; Molecular weight (SIZE): MW between 150 and 500 g/mol; Insolubility (INSOLU): log S less than 6; Flexibility (FLEX): no more than nine rotatable bonds; Saturation (INSATU): fraction of carbons in the sp3 hybridization not less than 0.25)] Based on these criteria, INSATU for cinnamaldehyde, that is, the fraction of carbons in the sp3 hybridization, violates the rule, and thus, the values cross the pink area. In the case of eugenol and cineol, all parameters stay within limits and thus support a good bioavailability, keeping in mind that eugenol has a little crossing of pink area.

In the log P method developed by Wildman and Crippen (WLOGP) vs. TPSA reference, the BOILED-Egg model (Daina and Zoete, 2016) agrees on an assessment of passive gastrointestinal absorption (HIA) and brain penetration (BBB) as a function of molecule position. The white zone denotes that passive absorption through the gastrointestinal system is likely, whereas the yellow region (yolk) indicates that brain penetration is likely. The yolk and white parts are not mutually exclusive. The points are additionally colored blue if they are expected to be actively effluxed by P-gp (PGP+) and red if they are expected to be a P-gp non-substrate (PGP). Considering this interpretation, cinnamaldehyde, eugenol, and cineol are effluxed by PGP- and have a high likelihood of brain penetration, as demonstrated by the BOILED-Egg model.

The evaluation of the ADMET properties of a medicinal drug is becoming crucially influential. The use of computational techniques has made determining ADMET characteristics of substances much easier. All the investigated drugs had TPSA values of less than 100, indicating good oral absorption or membrane permeability. Chemical absorption levels are often estimated using the Caco-2 permeability, intestinal absorption (human), skin permeability, and P-glycoprotein substrate or inhibitor. The tested molecule has a high Caco-2 permeability and is quickly absorbed when the Papp coefficient is larger than 8 × 10^−6^ and the anticipated value is greater than 0.90. The permeability of Caco-2 was high in all compounds. Anything less than 30% absorption in the human gut is termed inadequate absorption. In this case, all the substances tested exhibited a high absorption rate (>90%). Because a chemical with a log Kp > −2.5 has a relatively low skin permeability, cinnamaldehyde and cineol may have poor skin permeability, while eugenol and cineol may have excellent skin penetration. P-glycoprotein is a member of the ATP-binding transmembrane glycoprotein family [ATP-binding cassette (ABC)], which can excrete medicines from cells. Except for cineol, the ADMET data revealed that the compounds tested are neither substrates nor inhibitors of P-glycoprotein.

Drug distribution in tissues is primarily demonstrated by distribution volume at steady state (VDss), fraction unbound [human], CNS permeability, and blood–brain barrier membrane permeability in tissues (logBB). When VDss is less than 0.71 L kg^−1^, the distribution volume is thought to be quite low (log VDss = -0.15). When VDss is larger than 2.81 L kg^−1^ (log VDss> 0.45), the distribution volume is considered to be high. The distribution volumes of our tested substances were low.

In terms of permeability, compounds with logBB>0.3 are thought to flow through the blood–brain barrier with ease. Except for eugenol, the compounds tested have a logBB greater than 0.3, indicating that they may easily cross through the blood–brain barrier (logBB 0.185). Because logPS-3 is present in our tested drugs, they are unable to cross the CNS. Cytochrome P450 enzymes are very important for the metabolism of many drugs in the liver. This class is comprised of more than 50 enzymes, and CYP 1A2, 2C9, 2C19, 2D6, 2E1, and 3A4 with CYP3A4 and CYP2D6 possess a key role in drug metabolism (above 90%). Our results confirm that all tested four compounds were not substrates for CYP3A4 and CYP2D6. All tested compounds were inhibitors of CYP1A2. Thus, our tested molecules may be metabolized in the liver. Among the tested molecules, cineol has the highest total clearance. Regarding safety profiling, eugenol showed mild AMES toxicity, and all compounds were observed as sensitive to the skin. These findings clearly indicate that skin sensitization may occur; however, it mainly depends on the dose utilized and the degree of encapsulation in the typical formulation.

Based on the findings of our earlier investigations (Rafey et al., 2021), the cinnamon EO, cardamom EO, and clove EO were tested individually and mixed in defined ratios and then tested for their combined effect in the different combinations. In the individual case, the clove EO showed the highest MIC (0.024 mg/mL) against *S. epidermidis*, followed by the cinnamon EO (0.039 mg/mL). In the case of *S. aureus,* the cinnamon EO showed the highest inhibition (0.078 mg/mL), followed by the clove EO (0.097 mg/mL). Based on these findings, it was decided to further process only clove, cinnamon, and cardamom EOs to see their combined effect.

In the MIC assays of diverse combinations (as explained above), combination F2 comprising cinnamon and clove EOs was chosen for further activities because they showed excellent inhibition of *S. epidermidis* (0.0625/0.0312 mg/mL) and *S. aureus* (0.0156/0.0078 mg/mL). It was concluded that the EOs of clove and cinnamon may have a synergistic effect in the eradication of oral bacteria. A possible reason for promising activities may be the presence of eugenol and cinnamaldehyde, which have both bactericidal and bacteriostatic activities (Ali et al., 2005).

Quorum sensing is one of the major mechanisms for bacterial biofilm formation; thus, inhibition of quorum sensing can demonstrate that a compound has antibiofilm features. *Chromobacterium violaceum* is a biomarker strain for bacterial quorum sensing. In this experiment, different concentrations of essential oils were tested for anti-quorum sensing activities, and diverse concentrations of clove and cinnamon EOs were analyzed for inhibition of zones and violacein. The N2 formulation presented promising results and was processed further. The tested molecules were analyzed further for antibiofilm potential, and a strong antibiofilm activity was recorded for N2 and N3 against tested strains. It confirmed that biofilm inhibition was due to quorum sensing (Qaisrani et al., 2021).

## 5 Conclusion

The molecular dynamic and docking studies revealed that major components of essential oils were compatible with each other and the bio-composite polymer Carbapol 940. Further *in silico* characterization indicated that the nanocomposite components complied with the established parameters. Moreover, the combination of cinnamon and clove EOs showed significant antimicrobial, anti-quorum sensing, and antibiofilm activity against both the clinical oral strains *S. aureus* and *S. epidermidis.* These EOs are considered to have synergistic effects and will be considered for encapsulation in a nanocomposite dosage form. Thus, it was concluded that cinnamon and clove EO-based nanocomposite with the Carbapol 940 formulation could be further processed for oral formulation and dental material development due to strong stability and enhanced biological activities. Further investigations on the effect of copolymer addition and clinical investigations are suggested.

## Data Availability

The original contributions presented in the study are included in the article/Supplementary Material; further inquiries can be directed to the corresponding author.
