# Supplementary figures and images for: Exploring essential oil-based bio-composites: molecular docking and in vitro analysis for oral bacterial biofilm inhibition
Source: Front Chem. 2024 Jul 17;12:1383620. doi: 10.3389/fchem.2024.1383620 (PMC11288909; doi:10.3389/fchem.2024.1383620)

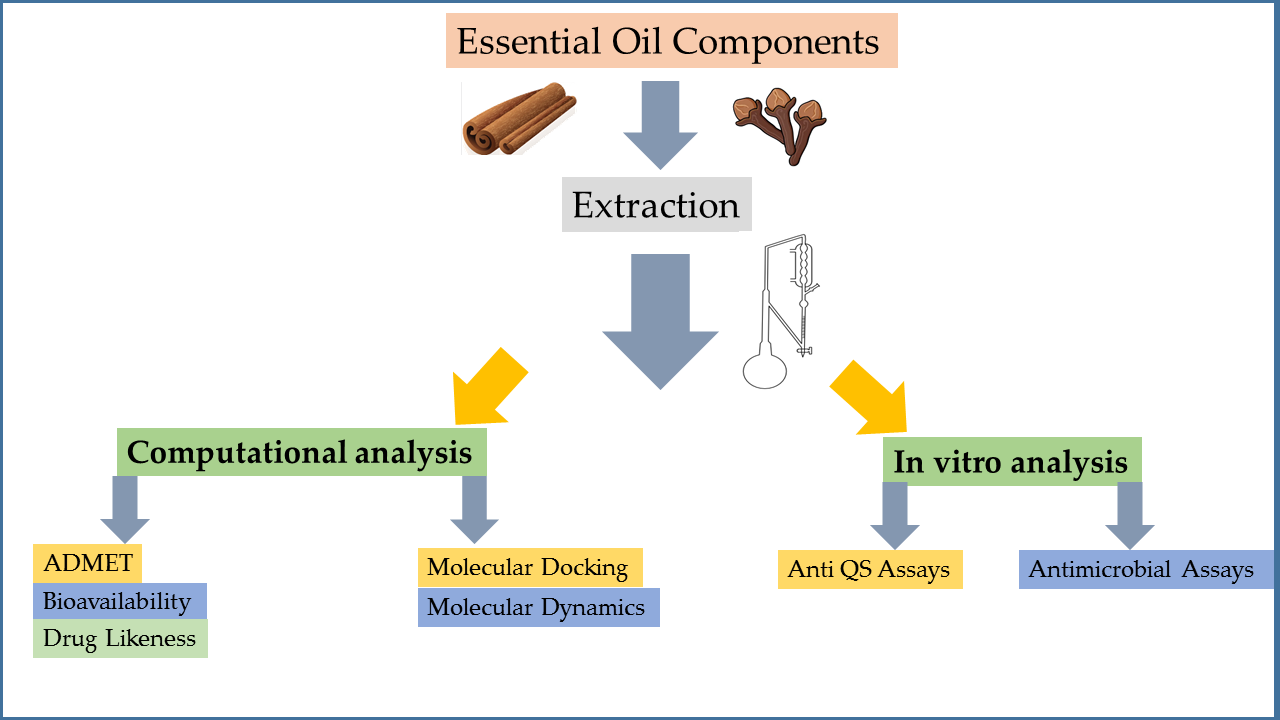

Supplement: Supplementary file 1 [file Image1.TIF]
